# The relationship between headache-attributed disability and lost productivity: 3 Attack frequency is the dominating variable

**DOI:** 10.1186/s10194-023-01546-9

**Published:** 2023-02-14

**Authors:** Andreas Husøy, Zaza Katsarava, Timothy J. Steiner

**Affiliations:** 1 Department of Neuromedicine and Movement Science, NTNU Norwegian University of Science and Technology, Edvard Griegs Gate 8, 7030 Trondheim, Norway; 2 Evangelical Hospital Unna, Unna, Germany; 3 Department of Neurology, University of Duisburg-Essen, Essen, Germany; 4 EVEX Medical Corporation, Tbilisi, Georgia; 5 Department of Neurology, University of Copenhagen, Copenhagen, Denmark; 6 Division of Brain Sciences, Imperial College London, London, UK

**Keywords:** Migraine, Disability, Headache frequency, Lost productivity, Association analysis, Health economics, Health policy, Migraine preventative drugs, Global Campaign against Headache

## Abstract

**Background:**

In an earlier paper, we examined the relationship between headache-attributed disability, measured as proportion of time in ictal state, and lost productivity. In a linear model, we found positive and significant associations with lost paid worktime, lost household worktime and total lost productivity (paid + household), but with high variance, which was increased when headache intensity was introduced as a factor. We speculated that analyses based on headache frequency alone as the independent variable, eliminating both the subjectivity of intensity estimates and the uncertainties of duration, might show stronger associations.

**Methods:**

Focusing on migraine, we used individual participant data from 16 countries surveyed either in population-based studies or in the Eurolight project. These data included frequency (headache days/month), usual attack duration (hours), usual headache intensity (“not bad”, “quite bad”, “very bad”) and lost productivity from paid and household work according to enquiries using the Headache-Attributed Lost Time (HALT) questionnaire. We used multiple linear regressions, calculating regression equations along with unstandardized and standardized regression coefficients. We made line and bar charts to visualize relationships.

**Results:**

Both frequency and intensity were significant predictors of lost productivity in all multiple linear regressions, but duration was a non-significant predictor in several of the regressions. Predicted productivity in paid work decreased among males by 0.75–0.85 days/3 months for each increase of 1 headache day/month, and among females by 0.34–0.53 days/3 months. In household chores, decreases in productivity for each added day/month of headache were more similar (0.67–0.87 days/3 months among males, 0.83–0.89 days/3 months among females). Visualizations showed that the impact of duration varied little across the range of 2–24 h. The standardized regression coefficients demonstrated that frequency was a much better predictor of lost productivity than intensity or duration.

**Conclusion:**

In the relationship between migraine-attributed impairment (symptom burden) and lost productivity, *frequency* (migraine days/month) is the dominating variable – more important than headache intensity and far more important than episode duration. This has major implications for current practice in headache care and for health policy and health-resource investment. Preventative drugs, grossly underutilized in current practice, offer a high prospect of economic benefit (cost-saving), but new preventative drugs are needed with better efficacy and/or tolerability.

## Background

Headache disorders are the cause of much population ill health, and the resultant disability has been revealed with increasing clarity over the last decade [1–10]. Also consequential is lost productivity, evidenced in multiple studies [for example, 11–16]. Lost productivity is of substantial economic importance [17–20], with explicit implications for health policy and investment of resources in headache services and care [21–23].

In an earlier paper, we used a number of approaches to examine the relationship between headache-attributed disability and lost productivity [24] (using, as we do here, the term *disability* in the sense applied in the Global Burden of Disease (GBD) study [2–8]). Focusing on migraine, the most disabling headache disorder at population level, we made use of individual participant data (IPD) from Global Campaign population-based studies conducted in six disparate countries and from the Eurolight project in another three countries [24]. Available symptom data from these studies included headache frequency and usual duration and intensity of headache. Other data included lost productive time from paid work and household chores. We estimated proportion of time in ictal state (pTIS) from frequency and duration, and disability as the product of pTIS and disability weight (DW) for the ictal state of migraine from GBD [25]. In a linear model, we found positive and significant associations with lost paid worktime, lost household worktime and total lost productivity (paid + household), but with low values of R^2^ (0–0.22) due to high variance.

In other papers, modelling the effects of theoretical reductions in disability achieved through interventions, and applying the regression equations for each country to the population mean migraine-attributed disability, we found *pro rata* recoveries of lost productivity in the range 16–56% [22, 23]. In other words, on average, depending on country, one unit reduction in disability would be expected to recover 0.16–0.56 units of lost productivity. We concluded that relief of disability through effective treatment of migraine would, in most countries and most economies, recover sufficient lost productivity for investment in structured headache services (SHS [21]) to be cost saving, not merely cost-effective [23]. This greatly strengthened the economic argument for SHS as a form of intervention [21], especially since, in the variation between countries, country-income level was not a factor [23].

While GBD uses the metric *years lived with disability* (YLDs), a product of prevalence, mean pTIS and DW [25], YLDs are a measure not strictly of disability but of lost health more broadly [26–28]. Since DW for the ictal state of migraine is a constant, in further analyses we introduced headache intensity as a factor, seeking a more nuanced assessment of individual health loss. In relating this product to lost productivity we found, merely, increased variance. We speculated that analyses based on headache frequency alone as the independent variable, eliminating both the subjectivity of intensity estimates and the uncertainties of duration, might show stronger associations [24].

This study, a project within the Global Campaign against Headache [29–32], accordingly investigates the individual contributions of frequency, duration and intensity of migraine attacks to lost productivity. There is a very important subtext: should it prove that frequency is the main driver of headache-attributed lost productivity, interventions would be better focused on attack prevention.

## Methods

### Data acquisition

We used IPD from Global Campaign population-based studies in eight disparate countries with large sample sizes (*N* > 1,000): China [33], Ethiopia [15], India [34], Nepal [14], Pakistan [35], Russia [36], Saudi Arabia [37] and Zambia [16]. We also used IPD from eight selected samples, from a further eight countries, surveyed in the Eurolight project [38, 39] (see below).

### Ethics

In all contributing studies, ethics approvals and consents had been obtained according to local requirements; these are reported in the respective publications [14–16, 33–39].

### Sampling and data collection in population-based studies

Data from these studies were collected using standardised methodology [40, 41], with any necessary adaptations again reported in the respective publications [14–16, 33–39].

Each study was a cross-sectional survey employing randomised cluster sampling to reflect the diversities of the country or area, thereby generating representative samples. The enquiry procedure in all countries except Saudi Arabia involved unannounced visits at random households (“cold-calling”) within each cluster. One adult member (18–65 years) of each household was randomly selected for interview. Cultural sensitivities in Saudi Arabia precluded such visits; here, therefore, the survey was conducted by random dialling of mobile phones [37].

All interviews used the Headache-Attributed Restriction, Disability, Social Handicap and Impaired Participation (HARDSHIP) questionnaire [41], translated into the local language(s) in accordance with the Global Campaign translation protocols [42]. HARDSHIP included demographic enquiry, a neutral headache screening question, diagnostic questions based on the International Classification of Headache Disorders (ICHD) [43] and enquiries into headache-attributed symptom burden and lost productive time.

### Sampling and data collection in Eurolight

The Eurolight project used a structured questionnaire that was a close derivative of HARDSHIP, sampling from ten countries of the European Union but with sampling methods that varied between countries. The detailed methods have been published elsewhere [38]. We used IPD only from eight of these countries (Austria, France, Germany, Italy, Lithuania, Luxembourg, Netherlands and Spain) with samples that were population-based or were derived from workplace or general (non-headache) clinical settings. We discarded those from Ireland and UK, and additional samples from Netherlands and Spain, that were generated by patient organisations [38].

### Symptom burden

Symptom enquiry included headache frequency, which was reported in the studies as headache days/month, not attacks/month. Usual attack duration was reported in minutes, hours or days. Usual headache intensity was reported as “not bad”, “quite bad” and “very bad”.

### Lost productivity

Enquiry into lost productive time during the preceding 3 months used the Headache-Attributed Lost Time (HALT-90) questionnaire [44] as a module within HARDSHIP [41]. Two questions (1 and 2) counted days in that period (i) completely missed from paid work (absenteeism) and (ii) with < 50% productivity (less than half achieved of what was normally expected) while at work (presenteeism), in each case because of headache. Two similar questions (3 and 4) asked for days of household work (iii) completely missed and (iv) with < 50% productivity [44].

### Analysis

#### Diagnosis

Diagnoses in all studies were made algorithmically, applying modified ICHD criteria [43]. For the analyses here, only participants with episodic migraine were of interest. These were identified, after exclusion of headache on ≥ 15 days/month, by first applying criteria for migraine, then those for tension-type headache, and finally those for probable migraine [40, 43]. Migraine and probable migraine were combined for further analyses.

### Statistics

We expressed all attack durations in hours and all attack frequencies in days per month. For intensity, we interpreted the response options as mild, moderate and severe, and converted these to a numerical scale of 1–3. We expressed lost productivity at individual level in accordance with responses to the four questions from HALT in whole days/3 months, equating, according to accepted methodology, “less than half achieved” to “nothing achieved” and counterbalancing this by equating “more than half achieved” to “everything achieved” [44]. We summarised the IPD as means with standard errors (SEMs) and/or standard deviations (SDs), or exact figures with percentages, as appropriate.

Multiple linear regressions based on IPD were calculated to predict lost productivity in both paid work and household chores. Frequency, duration and intensity of migraine attacks were included as predictors in the regression models. Separate analyses were performed on pooled data from Eurolight and those from the population-based studies, stratified by gender. Results from the regression models were reported as F-values, degrees of freedom, *p*-values and R^2^-values. In addition to calculating regression equations, with unstandardized regression coefficients (β = degree of change in the dependent variable for every unit of change in the independent variable), we also calculated standardized regression coefficients to facilitate direct comparisons, between the three predictors (frequency, duration, intensity), of impact on lost productivity. To uncover potential collinearity between any of the predictors, which would have introduced bias into the model, we calculated variance inflation factors (VIFs).

Line charts were made to visualize the relationships between lost productivity and frequency and duration of migraine attacks, and bar charts to visualize the relationship between lost productivity and intensity of migraine attacks.

We used Statistical Package for the Social Sciences (SPSS) version 26.0 for all analyses. We considered *p* < 0.05 to be significant.

## Results

Data were available from *N* = 5,048 participants with migraine in the population-based sample and from *N* = 2,752 in the Eurolight sample. Not all provided a complete set of responses required for these analyses: an account of missing data is in Table 1. In particular, among the population-based sample, one third (35.5%) of males did not provide responses for lost household days and one quarter (25.3%) of females did not do so for lost work days. These gender-based differences were not seen among the Eurolight sample, whose responder proportions were invariably higher (Table 1).

**Table 1:** Numbers of participants and of those with missing data for each variable

	**Population-based**		**Eurolight**	
	**Male**	**Female**	**Male**	**Female**
**N with migraine**	1,856	3,192	870	1,882
**Numbers missing data**				
Lost productivity				
HALT questions 1 + 2	136	664	42	172
HALT questions 3 + 4	578	310	46	115
Disability factors				
F only	17	10	2	2
D only	88	156	5	26
I only	4	5	3	17
F + D	5	10	1	4
F + I	0	0	0	1
D + I	2	1	3	10
F + D + I	0	0	0	1
Total with missing disability data of whom also missing HALT 1 + 2 data^a^ and of whom also missing HALT 3 + 4 data^a^	116	182	14	61
	11	37	2	18
	35	32	2	14
Final N in HALT 1 + 2 analyses^a^	1,615 (87.0%)	2,383 (74.7%)	816 (93.8%)	1,667 (88.6%)
	(1,856 – [136 + (116–11)])	(3,192 – [664 + (182–37)])	(870 – [42 + (14–2)])	(1,882 – [172 + (61–18)])
Final N in HALT 3 + 4 analyses^a^	1,197 (64.5%)	2,732 (85.6%)	812 (93.3%)	1,720 (91.4%)
	(1,856 – [578 + (116–35)])	(3,192 – [310 + (182–32)])	(870 – [46 + (14–2)])	(1,882 – [115 + (61–14)])

*HALT* Headache-Attributed Lost Time, *F* headache frequency, *D* headache duration, *I* headache intensity

^a^corrections applied to avoid double counting

### Descriptives

The regression analyses were performed on the IPD, but the data on lost productivity and attack frequency, duration and intensity are summarised in Table 2, stratified by gender and sample. Overall, medians were lower than means, indicating skewedness in the data. All SEMs were small, indicating that sample means were accurate estimates of the true population means. Females had migraine episodes more frequently than males, and, in the Eurolight sample, duration was longer for females than males. Intensity was similarly distributed between genders, but the proportions with “very bad” headache were greater in the population-based sample than in the Eurolight sample. Lost productivity in paid work was similar between males and females in the Eurolight sample (2.5 *vs* 2.7 days/3 months), whereas females in the population-based sample lost fewer days from paid work (2.2 days *vs* 3.3 days/3 months). Females in both samples reported greater losses than males from household chores (3.9–4.3 *vs* 2.8–3.0 days/3 months).

**Table 2:** Symptom burden (attack frequency, duration and intensity) and lost productivity (work days [HALT 1+2] and household days [HALT 3+4]) in the two samples

Sample	Frequency (days/month)	Duration (hours)	Intensity			HALT 1 +2 (days/3 months)	HALT 3 +4 (days/3 months)
	**mean ±SEM (median)**		**Not bad n (%)**	**Quite bad n (%)**	**Very bad n (%)**	**Mean ±SEM (median)**	
**Population-based**							
male	2.7 ±0.1 (2.0)	24.9 ±1.2 (6.0)	104 (5.6)	1012 (54.7)	734 (39.7)	3.3 ±0.2 (1.0)	3.0 ±0.2 (0.0)
female	3.2 ±0.1 (2.0)	26.7 ±0.9 (12.0)	162 (5.1)	1682 (52.8)	1342 (42.1)	2.2 ±0.1 (0.0)	3.9 ±0.2 (2.0)
**Eurolight**							
male	2.5 ±0.1 (1.7)	20.9 ±1.1 (8.0)	164 (19.0)	522 (60.4)	178 (20.6)	2.5 ±0.3 (0.0)	2.8 ±0.3 (0.0)
female	3.3 ±0.1 (2.5)	37.3 ±1.1 (24.0)	179 (9.7)	1138 (61.4)	536 (28.9)	2.7 ±0.1 (0.0)	4.3 ±0.2 (2.0)

*HALT* Headache-Attributed Lost Time, *SEM* standard error of mean

### visualizations

**Fig. 1:**
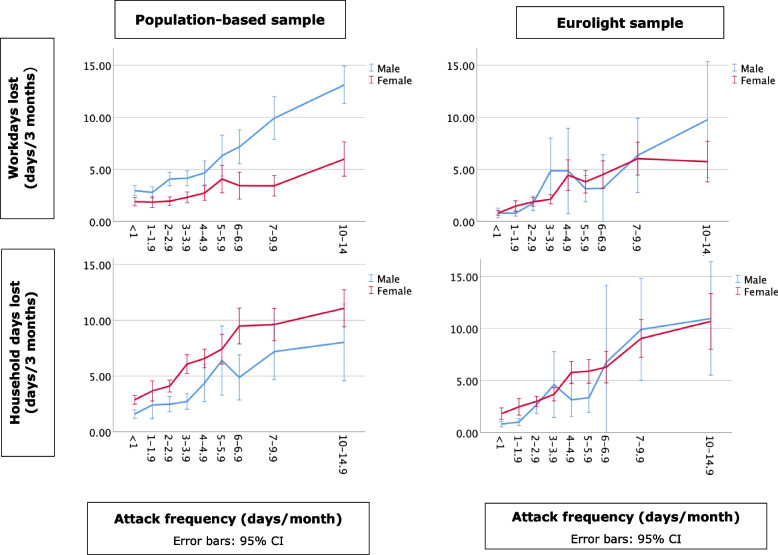
Relationship between lost productivity (work days and household days) and attack frequency by sample and gender

**Fig. 2:**
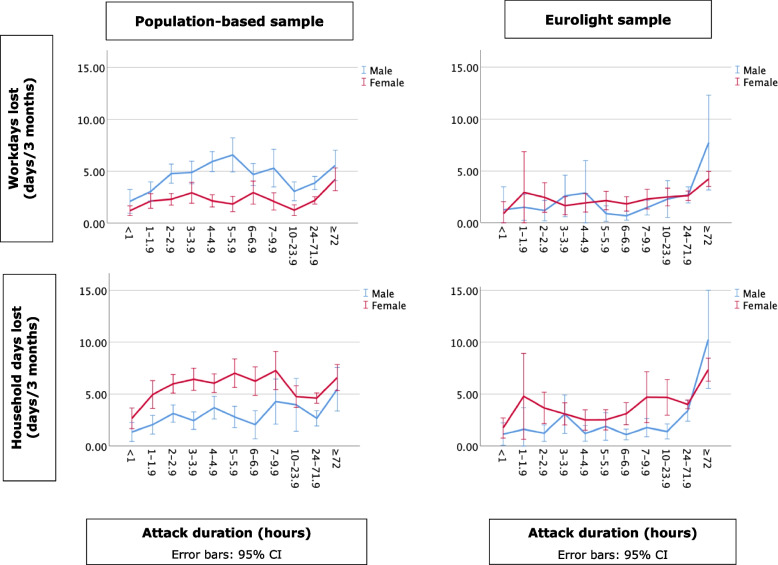
Relationship between lost productivity (work days and household days) and attack duration by sample and gender. The Y axis is on the same scale as in Fig. 1 for ease of comparison

**Fig. 3:**
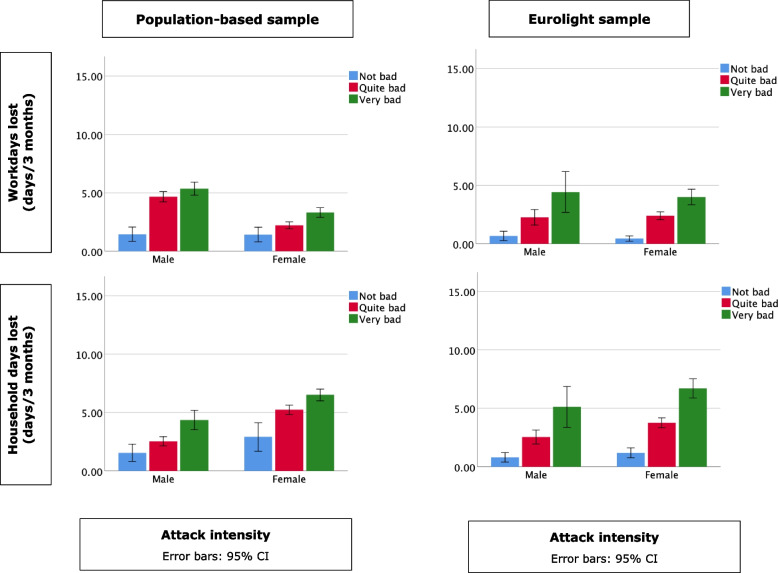
Relationship between lost productivity (work days and household days) and attack intensity by sample and gender. The Y axis is on the same scale as in Fig. 1 for ease of comparison

In Figs. 1, 2 and 3, frequency, duration and intensity of migraine attacks are plotted against lost productivity in paid work (lost work days) and household chores (lost household days). No direct statistical tests were performed, but the visualizations clearly show positive linear relationships between frequency and intensity on the one hand and lost productivity on the other in all groups. Duration had no such relationship: attacks reportedly lasting from two to 24 h were associated with very similar productivity losses, with small up-kicks at the far-right indicative of impacted productivity on the next day from attacks of > 24 h’ duration. In the population-based sample, headache had greater impact on productivity in paid work in males than in females, and the opposite in household chores. Gender differences were small or none in the Eurolight sample.

### Multiple linear regressions

Multiple linear regressions were performed on the IPD to predict lost productivity from attack frequency, duration and intensity (Table 3). Values of R^2^ were small, and ranged from 0.05 to 0.16 because of high variance, but all regression models were significant (*p* < 0.001). Therefore, it was possible to use the equations to predict productivity losses at population level. The VIFs were small (< 1.08), indicating no collinearity between the predictors.

**Table 3:** Multiple linear regressions predicting lost productivity (work days and household days) from frequency, duration and intensity of migraine attacks

Sample	N	Regression model	Equation (unstandardized coefficients)	Standardized coefficients			VIF	p		
				**F**	**D**	**I**		**F**	**D**	**I**
	**HALT questions 1+ 2 (lost work days per 3 months)**									
**Population-based**										
male	1,615	F (3, 1611) = 94.3 *p* < 0.001, R^2^ = 0.15	Y = 0.85xF + 0.01xD + 1.30xI – 0.93	0.37	0.03	0.11	<1.04	<0.001	0.17	<0.001
female	2,383	F (3, 2379) = 38.2 *p* < 0.001, R^2^ = 0.05	Y = 0.34xF + 0.01xD + 0.91xI – 1.05	0.18	0.06	0.10	<1.03	<0.001	0.007	<0.001
**Eurolight**										
male	816	F (3, 812) = 27.8 *p* < 0.001, R^2^ = 0.09	Y = 0.75xF + 0.02xD +1.32xI – 2.46	0.26	0.06	0.10	<1.05	<0.001	0.06	0.003
female	1,667	F (3, 1663) = 63.0, *p* < 0.001 R^2^ = 0.10	Y = 0.53xF + 0.01xD + 1.31xI – 2.14	0.26	0.04	0.13	<1.07	<0.001	0.14	<0.001
	**HALT questions 3 + 4 (lost household days per 3 months)**									
**Population-based**										
male	1,197	F (3, 1193) =34.9 *p* < 0.001, R^2^ = 0.08	Y = 0.67xF + 0.01xD + 1.15xI – 1.77	0.25	0.07	0.10	<1.05	<0.001	0.01	0.001
female	2,732	F (3, 2728) =122.2 *p* < 0.001, R^2^ = 0.12	Y = 0.89xF + 0.01xD + 1.43xI – 0.98	0.33	0.03	0.10	<1.03	<0.001	0.14	<0.001
**Eurolight**										
male	812	F (3, 808) =52.5 *p* < 0.001, R^2^ = 0.16	Y = 0.87xF + 0.04xD + 1.31xI – 2.91	0.31	0.16	0.11	<1.05	<0.001	<0.001	0.001
female	1,720	F (3, 1716) =112.2 *p* < 0.001, R^2^ = 0.16	Y = 0.83xF + 0.02xD + 2.07xI – 3.53	0.32	0.10	0.16	<1.08	<0.001	<0.001	<0.001

*F* frequency of migraine attacks (continuous, measured in days/month), *D* duration of migraine attacks (continuous, measured in hours), *I* intensity of migraine attacks (ordinal: 1=“not bad”; 2=“quite bad”; 3=“very bad], *VIF* variance inflation factor

### Lost productivity in paid work

Both frequency and intensity of migraine attacks were significant predictors of lost productivity in paid work in males, whereas duration was not: in the population-based sample, predicted productivity in paid work decreased by 0.85 days/3 months for each marginal increase of 1 headache day/month and by 1.3 days/3 months for each one-step increment in intensity, but by only 0.01 days/3 months for each marginal increase of 1 h in duration (unstandardized coefficients: Table 3). Findings were similar in the Eurolight sample. The standardized regression coefficients showed that frequency was a much better predictor of lost productivity in paid work than intensity or duration (Table 3).

There were some gender-related differences. While results were mostly similar between males and females in the Eurolight sample, each marginal increase of 1 headache day/month led to a slightly greater decrease in productivity in males than in females (0.75 *vs* 0.53 days/3 months). In the population-based sample, frequency was a much more important predictor of lost productivity in males than in females (0.85 *vs* 0.34 days/3 months). Furthermore, duration was a significant predictor for lost productivity in paid work in females but not in males.

### Lost productivity in household chores

As in paid work, the standardized regression coefficients demonstrated frequency to be the best predictor by far of lost productivity in household chores in both genders (Table 3). Frequency, duration and intensity of migraine attacks were all significant predictors of lost productivity in household chores in males. This was also true for females in the Eurolight sample, whereas only frequency and intensity were significant among females in the population-based sample. Similar regression coefficients for frequency were found for both genders in the Eurolight sample and for females in the population-based sample: each marginal increase of 1 headache day/month led to decreased productivity in the range of 0.83–0.89 days/3 months. Impact of frequency was somewhat less among males in the population-based sample (0.67 days/3 months).

Overall, the standardized and unstandardized regression coefficients for duration and intensity were quite similar between the different regression equations (Table 3). Frequency on the other hand, was more important in predicting household losses than those from paid work among females, whereas the opposite was true for males in the population-based sample.

## Discussion

This was the third in a series of studies examining the relationship between symptom burden of migraine and lost productivity. Factors considered were frequency, duration and intensity of migraine attacks. In our analyses, very small VIF values indicated no collinearity between these. In summary, both frequency and intensity were significant predictors of lost productivity in all multiple linear regressions. Graphic visualizations showed linear relationships in both genders between frequency and lost productivity from both paid and household work. Relationships between intensity and lost productivity were more or less linear in both genders. However, the impact of duration varied little across the range of 2–24 h, increasing only (and as might be expected) when duration exceeded 24 h, with episodes presumably persisting into a second day. The salient finding, from standardized coefficients, was that frequency was by far the most important predictor of lost productivity. Intensity of attacks was substantially less important, and duration least so (not significantly in several of the analyses).

Gender differences tended to reflect stereotypical gender roles: migraine in females had greater impact on lost household than paid work. This was especially so in the population-based sample, which, in contrast to the Eurolight sample, and with the exception of Russia, was derived wholly from non-European countries. While responder proportions to all questions were generally high (in most cases > 85%), these gender roles probably accounted for the gender-based differences among the population-based sample, but not the European, in missing lost-productivity data. At issue was perceived irrelevance, with males rather less likely than females to report lost household days, and females rather less likely than males to be at work.

### Implications for headache services and care, and health policy

*Lifting The Burden* (LTB), conducting the Global Campaign against Headache, has promoted structured headache services (SHS) as the equitable and efficient health-care solution to headache [21], and derived evidence of their cost-effectiveness from theoretical economic analytical modelling [22, 23, 45]. Inclusion of indirect (lost productivity) costs in economic modelling has a profound effect, since these costs are about 90% of total costs attributed to migraine [20]. The potential for effective care to recover lost productivity as a consequence of symptom-burden reduction holds out the prospect that investment in care will be *cost saving* (costs regained at societal level exceed input costs) [23, 46]. Clearly, the relationship between symptom burden and lost productivity is central to the economics of headache care.

Our previous paper found a linear association between pTIS and lost productivity, with projected *pro rata* recovery of the latter in the range 0.16–0.56 units per unit reduction in the former [24]. Our analyses here demonstrate that this association is driven by *frequency* rather than duration.

This may not be surprising. An episode of migraine tends to disrupt the day even when relatively short-lasting, or truncated by acute therapy. Planned tasks for the day are often cancelled early on. Furthermore, function for the remainder of the day is not always fully restored even after symptom remission. Only when symptoms carry over into a second day is there further impact on productivity. No such considerations apply to frequency.

The implications are very clear. Acute and preventative treatments each have important roles in mitigating symptom burden [42] and restoring lost health [23]. Both will reduce pTIS, with effects that are complementary. But recovery of lost productivity, and the associated economic gains, are substantially more likely with preventative medication (reducing days with migraine) than with acute treatment (reducing duration and/or intensity). It is possible to perform calculations directly on the projected economic benefits: for example, in males (according to the population-based data), 1 migraine day/month (prevented) equates to 0.85 days/3 months of lost productivity from paid work (averted) – a *pro rata* recovery of about 28%.

This is well above the 20% threshold estimated previously for care delivered by SHS to be cost saving [23].

### Migraine preventatives are greatly underutilized

Several epidemiological studies have demonstrated poor usage of preventative treatment among migraine sufferers. In the Eurolight studies, only 1.6–13.7% of those with migraine (varying by country) used preventative medications, despite much higher numbers likely to be eligible [47]. Similar proportions (7.9–13.0%) were earlier noted in samples from the United States of America [48, 49]. These were cross-sectional data. One longitudinal study from Germany showed that only 29% of migraine *patients* (according to claims data) used one or more preventatives over 9 years (2008 to 2016) [50] (the emphasis is important here: patients are, by definition, receiving medical care). These studies all reflected practice in high-income countries. The literature is sparse on the usage of preventatives in low-to-middle-income countries, but it is likely to be much lower, and such evidence as there is supports this [46, 51].

### Care practice and priorities must change

Failure to make full use of available preventative drugs, many of which are low cost and at least reasonably effective [42], is inexplicable and unjustifiable. It is true that many people with migraine who might benefit do not consult physicians [52], who, in most countries, must prescribe preventative drugs. It is true, also, that patients are resistant to taking daily medication for intermittent symptoms [52], and there is good empirical evidence that adherence is poor when preventative drugs are used [53, 54]. But the evidence suggests that much of the problem stems from reluctant prescribing by physicians [48, 49, 55–57]. To the extent that these disinclinations are unwarranted, the solution in all cases lies in educational initiatives such as those encompassed within SHS [21].

It is, however, also true that currently available preventative drugs are far from perfect. Their efficacy is limited, and none are free from side-effects. The evidence from this paper swings the pharmacoeconomic argument in favour of the current focus on development of new preventative drugs.

### Strengths and limitations

A major study strength was the utilisation of IPD from 16 studies conducted in very disparate countries, with total *N* = 7,800. Many of these studies used similar methodology [40, 41], although most of the Eurolight data were not population-based [38] and were separately analysed for this reason. The limitations were those inherent in data dependent on subjective evaluation and recall, these being unavoidable.

## Conclusion

In the relationship between symptom burden of migraine and lost productivity, *frequency* (migraine days/month) is the dominating variable – more important than headache intensity and far more important than episode duration. Accordingly, reduction in attack frequency offers greater potential for benefit than acute therapy, if benefit is ultimately measured in regained productivity. These, of course, are not alternatives: when indicated, preventative medication supplements acute therapy. In current practice, however, preventative drugs are grossly underutilized, and change must be promoted through educational initiatives. Furthermore, the economic considerations lend strong support to the development of new preventative drugs – so long as these provide better efficacy and/or tolerability.

## Data Availability

Not applicable.

## Abbreviations

DW: Disability weight
GBD: Global Burden of Disease (study)
HALT: Headache-attributed lost time (indices)
HARDSHIP: Headache-Attributed Restriction, Disability, Social Handicap and Impaired Participation (questionnaire)
ICHD: International Classification of Headache Disorders
IPD: Individual-participant data
LTB: *Lifting The Burden*
pTIS: Proportion of time in ictal state
SD: Standard deviation
SEM: Standard error (of mean)
SHS: Structured headache services
VIF: Variance inflation factor
WHO: World Health Organization
YLD: Year lived with disability
